# Morphological characteristics of the surgical neck region in the proximal humerus at different ages

**DOI:** 10.1186/s40001-022-00724-w

**Published:** 2022-06-30

**Authors:** Jialiang Guo, Yali Zhou, Meishuang Shang, Wei Chen, Zhiyong Hou, Yingze Zhang, Weichong Dong

**Affiliations:** 1 The School of Medicine, Nankai University, Tianjin, People’s Republic of China; 2 Department of Orthopaedics, The Third Hospital of Hebei Medical University, Shijiazhuang, People’s Republic of China; 3 Chinese Academy of Engineering, Beijing, People’s Republic of China; 4 NHC Key Laboratory of Intelligent Orthopeadic Equipment (The Third Hospital of Hebei Medical University), Shijiazhuang, People’s Republic of China; 5 Department of Pharmacy, The Second Hospital of Hebei Medical University, Shijiazhuang, People’s Republic of China

**Keywords:** Surgical neck region, Cortical thickness, Epiphyseal plate

## Abstract

**Background:**

The objective of the study was to demonstrate the cortical thickness character in the humeral surgical neck region using 3D cortical bone mapping technique and try to illustrate its morphological changes with age.

**Material and methods:**

Normal individuals, including 11 volunteers younger than 18 years, 87 adult men and 46 adult women, were enrolled. The cortical thickness and height of the surgical neck region was measured with Mimic and 3 Matic software. The height of the region was compared and measured. People with an age of 18–30 years was identified as Group I, people in 31–40 years as Group II, people in 41–50 years as Group III, people in 51–60 years as Group IV, and Group V including people ≥ 61 years.

**Results:**

Compared with the baseline Group I, cortical thickness was significantly decreased by 0.52 mm (*P* = 0.006) in Group III, by 0.76 mm (*P* < 0.001) in Group IV, and by 0.77 mm (*P* < 0.001) in Group V. Age moderately predicted cortical thickness with *r* = −0.5481. The height of the cortical change region was significantly decreased by 2.25 mm (*P* = 0.007) in Group II, by 2.98 mm (*P* < 0.001) in Group III, and by 2.07 mm (*P* = 0.02) in Group IV. However, no significant decrease was illustrated in Group V (0.57 mm) (*P* = 0.891). The relation between age and the height of the cortical thickness change region was nonlinear.

**Conclusions:**

This study identified an obvious decrease in cortical thickness with aging, and the height was curve fitted with aging in surgical neck region.

## Introduction

Proximal humeral fracture (PHF) affects patient health and is the third most common fracture in the elderly [1, 2]. As population demographics continue to change, the incidence of proximal humeral fracture is expected to increase by 50% in 2030 [3]. Osteoporotic fractures are characterized by loss of bone mass and deterioration of bone microarchitecture and have put a great burden on medical expenses [4, 5]. More and more evidences have demonstrated that focal structural weakness might result patients to fragility fractures [6, 7].

It was reported that PHF was caused by impact or muscular pull to the cortical weak region [8]. Many studies have focused on trabecular bone with obvious changes in its structure in osteoporotic disease. Nevertheless, cortical bone, not trabeculae, represents the majority of the mass of the skeletal system [5, 9]. Many recent studies have also reported that bone loss in the radius, femur, and tibia involves cortical bone [10–12]. Many anatomical locations and developmental or regional distributions of the bone have been assessed, and the results illustrate that the porosity and thickness of cortical bone, has a great impact on the loss of bone mass, fracture risk [13–16].

The measurements of cortical bone thickness are studied at many anatomic sites using radiographs or high-resolution peripheral quantitative computerized tomography. As technology advances, Mimics image processing software is also considered a useful method to illustrate the cortical thickness in different anatomic regions [17]. Researches of humerus have demonstrated good results when using cortical bone thickness, and it exhibited that there was an obvious correlation in radiographic cortical bone thickness and local mineral content [18].

Evidence indicates that the cortex is an essential factor in osteoporotic fracture risks, little information is available for the surgical neck region (SNR), which is always considered a weak position. There are a few researches which have analyzed trabecular bone remodeling of proximal humerus, but none of them addressed the problem of cortical thickness changes with Mimics in SNR. Furthermore, a precise definition of the SNR is lacking. The study was to demonstrate the cortical thickness of the humeral SNR using 3D cortical bone mapping and to illustrate its morphological changes with age. We also assessed the relationship between the SNR and proximal epiphyseal plate and formalized precise notation of the SNR.

## Material and methods

In the study (NCT04523415), the relevant data of subjects were evaluated retrospectively from 2015.1 to 2019.12. Ethical approval was agreed and obtained from the Regional Ethics Committee of our hospital, and the study was also guaranteed to conduct in accordance with the Declaration of Helsinki. Informed written consent was obtained from patients or their guardian in this study.

Normal individuals with complete radiography and CT data of shoulder were included. Normal healthy volunteers, including 87 men and 46 women without fractures, were collected and then measured the cortical thickness and height of the SNR. All subjects with pathological fractures (tumor, neurological disease) were excluded. Normal healthy volunteers were scanned with computerized tomography machine, and the image matrix size was same (1 mm slices). The threshold values were adjusted and optimized according to the density histograms of the patients.

**Fig. 1:**
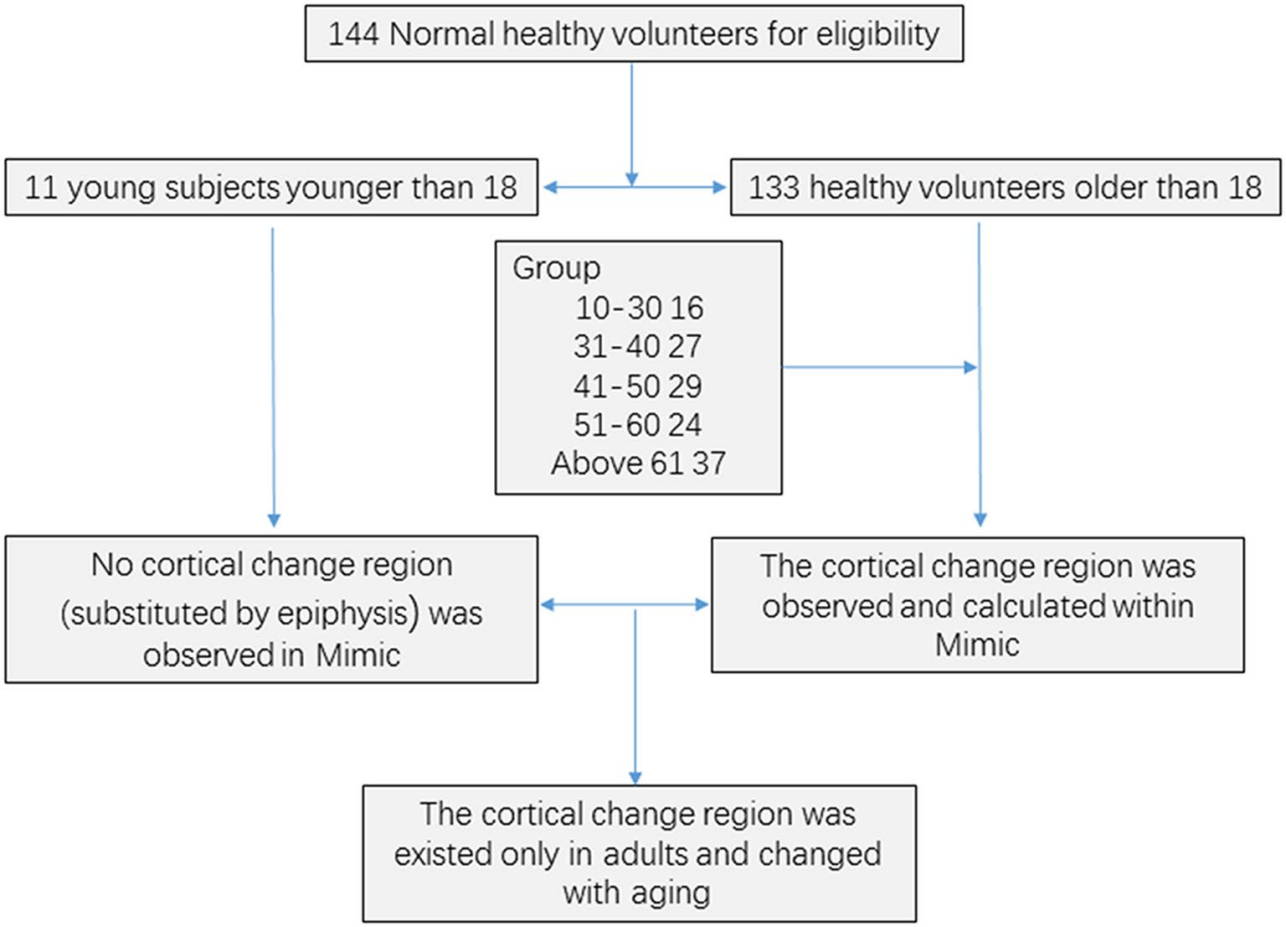
A flowchart about the enrolled volunteers in this research

The subjects enrolled were divided into five different groups: Group I = 18–30 years, Group II = 31–40 years, Group III = 41–50 years, Group IV = 51–60 years, and Group V ≥ 61 years. Individuals younger than 30 years was identified as Group I, and named as the baseline group, and compared with other 4 groups. Subjects younger than 18 years were collected to observe the morphology of the epiphyseal plate (Fig. 1).

### Proximal humeral cortical thickness map construction

Normal individuals, including 87 adults men (46.2 ± 13.3 years) and 46 adults women (53.6 ± 16.4 years), were enrolled and then measured the cortical thickness and height of the SNR. 11 subjects younger than 18 years was also collected to observe the morphology of epiphyseal plate. The DICOM images were downloaded through PACS and then were reconstructed to identify the fracture pattern. The data were then constructed with Mimics (21.0). In this study, Hounsfield units of 1600 (maximum) and 226 (minimum) were identified as the upper and lower threshold of the bone.

The 3D proximal humerus images were obtained, and exported directly to 3 Matic (12.0). The cortical thickness was measured with the function of the wall thickness analysis tool. The minimum threshold was identified as 0.33 mm, and the maximum thickness was 10 mm. The cortical thickness can be examined and observed integrally from the constructed cortical thickness map, and its relationship with SNR is also demonstrated.

### Cortical thickness and height

**Fig. 2:**
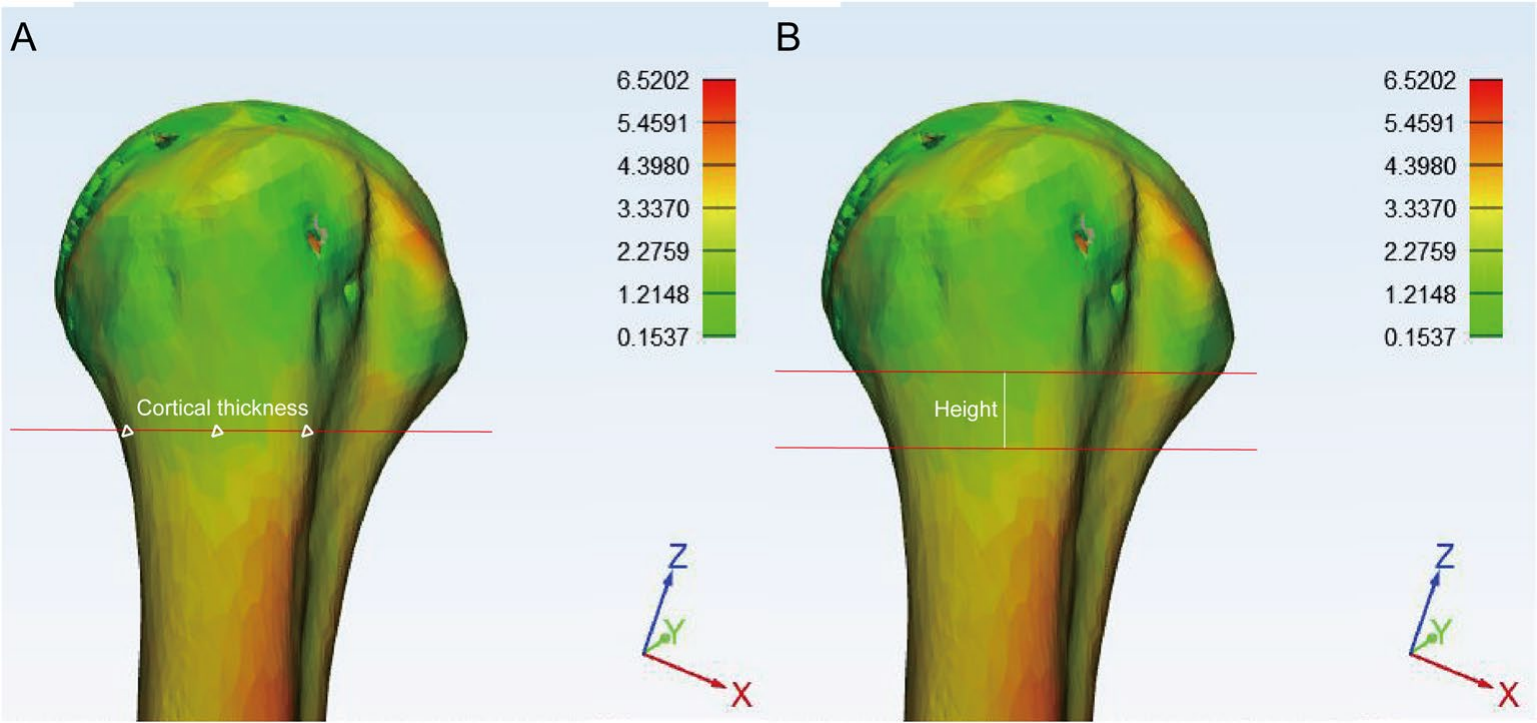
Parameter measurement method in the cortical change region. **A** The line around at the base of the lesser tuberosity was chosen as the plane to measure the cortical thickness of the surgical neck region. Three points were selected adjacent to the bicipital groove (anterolateral), middle of the cortical change region and posterolateral point. **B** The valid height of the cortical change region was measured. Valid height represents the minimum height of the cortical change region with same color (cortical thickness) in this 3D bone map

The line located immediately below the lesser tuberosity was selected to calculate the cortical thickness. Three different points were chosen: adjacent to the bicipital groove (anterolateral), middle of the greater tuberosity and posterolateral point (Fig. 2). The valid height which was identified as the distance between the proximal and distal directions) of SNR was also compared (Fig. 2). Valid height represents the minimum height of the cortical change region with same color (cortical thickness) in this 3D bone map.

### Statistical analysis

Relevant data are presented as the means ± standard deviation. Comparisons between the age groups were performed by one-way ANOVA with the S–N–K test, and Tukey test was utilized to determine the significant differences among different groups. For all analyses in this research, significance was identified as *P* < 0.05 level. All analyses were conducted using SPSS 22.0. Spearman correlations were calculated, and linear or nonlinear correlation coefficients were used and calculated to assess the correlation between ages and other parameters. The following correlation parameters were established. When |*r*| ≥ 0.8, it means a high correlation was noted. For 0.5 ≤ |*r|* < 0.8 means a moderate correlation. For 0.3 ≤ |*r*| < 0.5, it means a low correlation. When |*r*| < 0.3, there was no correlation in two variables.

According to the general requirements of statistics, taking *α* = 0.05, *β* = 0.1, the pre-experiment illustrate that the mean ± standard deviation of the height of the epiphyseal plate (the main index) in five different age groups was 7.89 ± 0.55 mm, 5.03 ± 0.53 mm, 5.01 ± 0.39 mm, 5.39 ± 0.65 mm, 7.03 ± 0.65 mm. The above parameters were substituted into PASS11 software, and concluded that the minimum number of cases to be completed in each group is 15 cases.

## Results

There was no cortical change region (substituted by epiphysis) in subjects younger than 18 years. The grown subjects were divided depending on different ages into 5 groups as follows: Group I was identified as the baseline group and included 16 normal individuals between 18 and 30 years of age with a mean age of 26.1 ± 3.6 years. Group II included 27 normal individuals between 31 and 40 years old with an average age of 34.9 ± 2.8 years. Group III included 29 normal individuals between 41 and 50 years with a mean age of 45.4 ± 3.1 years. Group IV included 24 normal individuals between 51 and 60 years with a mean age of 55.8 ± 3.0 years. 37 normal individuals ≥ 61 years old with a mean age of 66.9 ± 5.1 years was included Group V (Table 1).

**Table 1:** Cortical thickness and height about the cortical change region for different age groups [mean (95% CI)]

Groups (*m*/*f*)	Cortical thickness (mm)	Height (mm)	Age (years)
19–30 (*m* = 10; *f* = 8)	2.85 (2.67, 3.04)	8.02 (6.75, 9.29)	26.1 ± 3.6
31–40 (*m* = 21; *f* = 6)	2.48 (2.32, 2.64)	5.77 (4.82, 6.72)	34.9 ± 2.8
41–50 (*m* = 25; *f* = 4)	2.33 (2.07, 2.58)	5.04 (4.27, 5.82)	45.4 ± 3.1
51–60 (*m* = 15; *f* = 9)	2.09 (1.90, 2.22)	5.95 (5.13, 6.75)	55.8 ± 3.0
Above 60 (*m* = 16; *f* = 21)	2.08 (2.22, 2.40)	7.45 (6.86, 8.05)	66.9 ± 5.1

### Cortical thickness measurement of the cortical change region

**Fig. 3:**
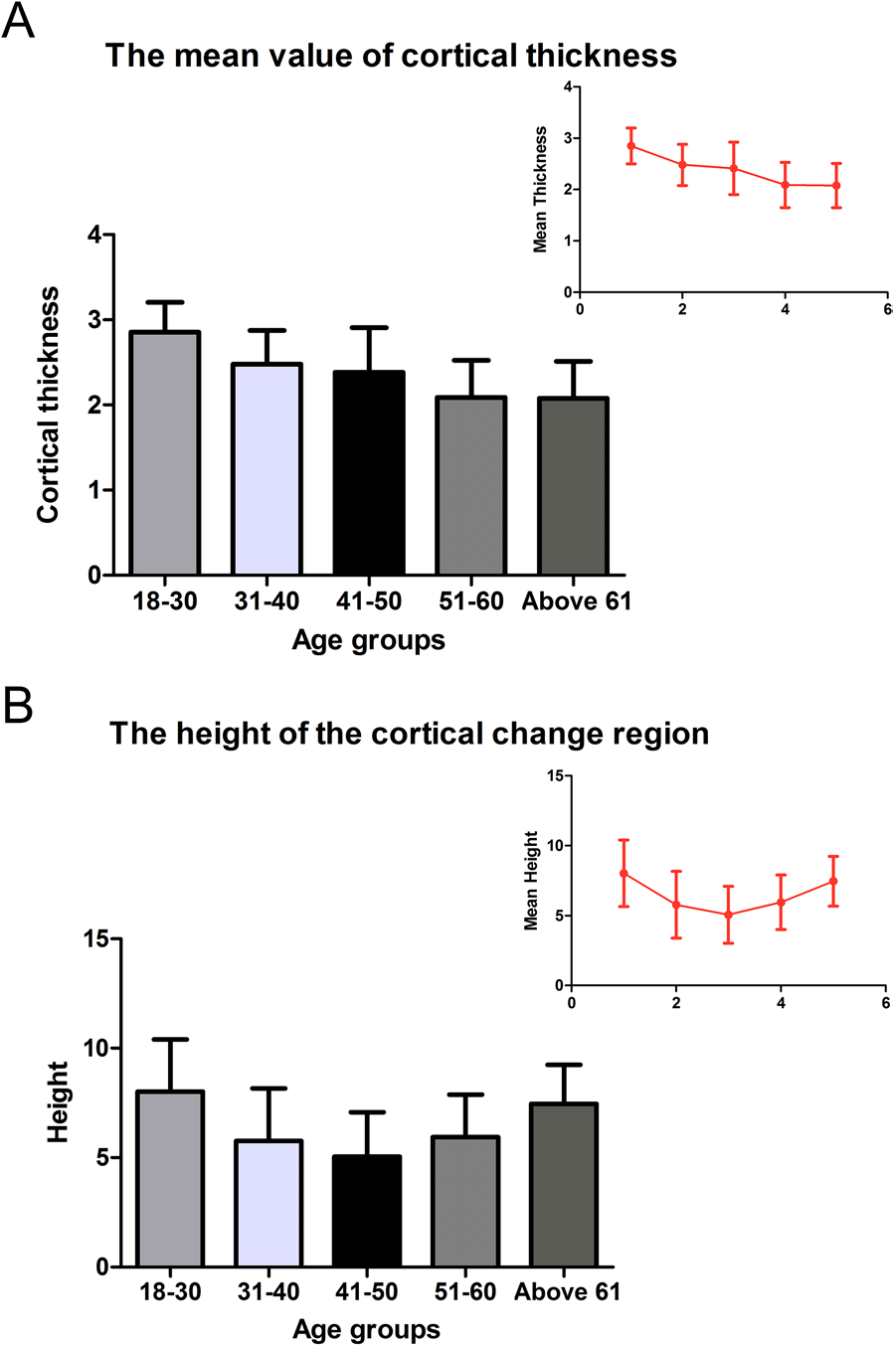
Measurement results of the surgical neck region thickness and height. **A** The column diagram and line chart of cortical thickness results. **B** Column diagram and line chart of the height measurement results

**Fig. 4:**
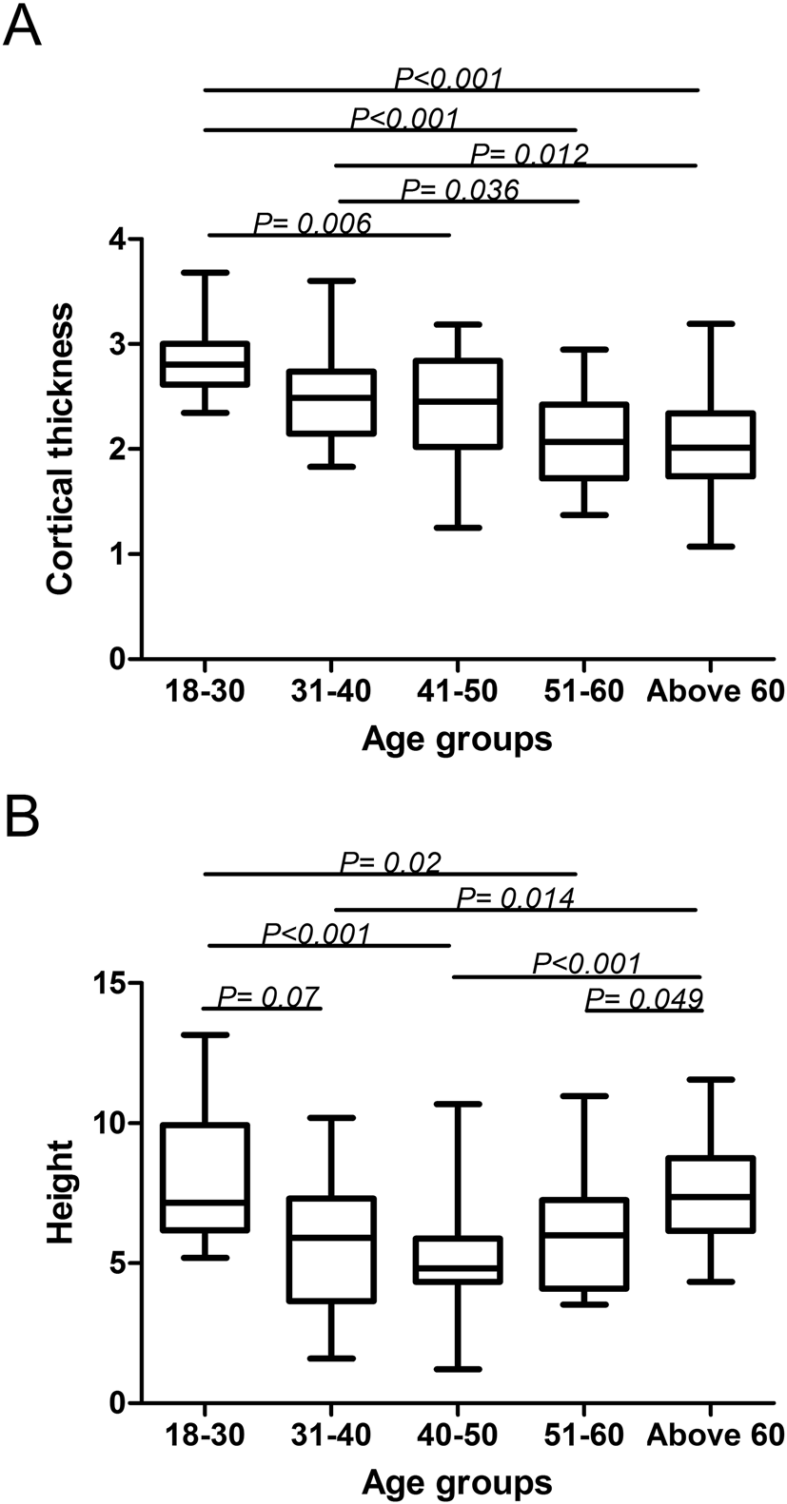
Cortical change region parameters in different age groups. **A** Box plot of cortical thickness comparison in different groups. The results show a significant difference between the age groups 18–30 and 41–100 years. **B** The box plot of valid height in different groups. All results illustrate a significant difference between the age groups 18–30 and 31–60 years

**Fig. 5:**
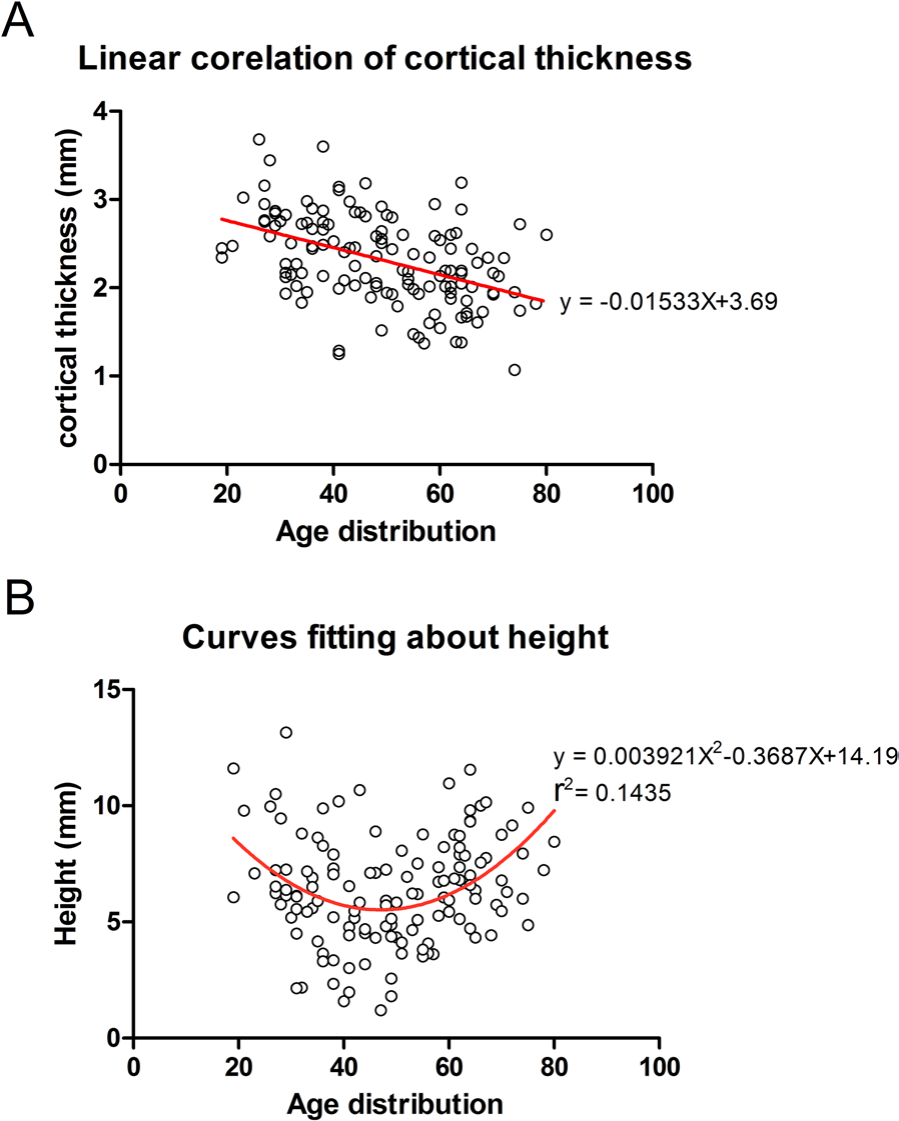
Correlation regression analysis between age and other parameters. **A** Bone cortical thickness was significantly linearly correlated with age (*P* < 0.0001). **B** The valid height was a significant curve fitted with age (*P* < 0.001). *P*-values determined for the whole collective

Compared with the baseline Group (Group I), the cortical thickness in Group II (31–40 years) was decreased by 0.37 mm (the CI was 2.32–2.64, − 13%) (*P* = 0.104), but the result was not significant. Cortical thickness was significantly decreased by 0.52 mm (the CI was 2.07–2.58, − 18.3%) (*P* = 0.006) in Group III (40–50 years), by 0.76 mm (the CI was 1.90–2.22, − 26.7%) (*P* < 0.001) in Group IV (51–60 years), and by 0.77 mm (the CI was 2.22–2.40, − 27%) (*P* < 0.001) in Group V (above 60 years) (Figs. 3A, 4A). Age moderately predicted cortical thickness with *r* = − 0.5481, and the linear correlation equation was as follows: *y* = − 0.01533*X* + 3.69 (Fig. 5A).

### The height measurement of the cortical change region

Compared with the baseline Group I, the height of the cortical change region was significantly decreased by 2.25 mm (CI 4.82–6.72, − 28.1%) (*P* = 0.007) in Group II (31–40 years), by 2.98 mm (CI 4.27–5.82, − 37.2%) (*P* < 0.001) in Group III (41–50 years), and by 2.07 mm (CI 5.13–6.75, − 25.8%) (*P* = 0.02) in Group IV (51–60 years). However, no significant decrease was illustrated in Group V (0.57 mm) (CI 6.86–8.05, − 7.1%) (*P* = 0.891) (Figs. 3B, 4B). The relation between age and the height of the cortical change region was nonlinear, and the nonlinear regression equation is as follows: *y* = 0.003921*X*^2^− 0.3687*X* + 14.19 (Fig. 5B).

## Discussion

Trabecular bone loss is a characteristic of osteoporosis and has promoted research and thinking into the structural basis of bone fragility in recent decades. Approximately 70% of appendicular bone loss originates from cortices, and the main bone loss occurs by intracortical remodeling that cavitates the cortex [12, 15]. The present study is the first of its kind to illustrate the cortical thickness at various ages in proximal humerus within a 3D bone cortical thickness map. It was illustrated that the SNR had a thinner cortical thickness and decreased with aging. The height of the SNR was curve fitted with different ages and was still observed across adolescent ages. The phenomenon of cortical height reduction in Group I, II, III was possibly caused by delayed maturation of epiphyseal plate which was still not studied in previous research. It was concluded that the SNR was a region that exhibited a different cortical thickness compared with the adjacent structure as well as demonstrated a potential relationship with or even originated from the closed epiphyseal plate in adolescents which need to be testified with further study.

Loss of cancellous and cortical bone are reported to be the causes of age-related proximal humeral fractures, but bone loss mainly involves cortical bone given larger accessible areas for bone resorption in cortical versus trabecular bone [12]. Therefore, cortical bone has a great impact on the loss of bone mass and fracture risk [14]. It was reported that the associations between trabecular microstructure and prevalence of fracture were no longer apparent after adjustments for aBMD in older men with fractures [14]. Previous work has figured out that it is necessary to investigate the changes in cortical bone histological structure of femoral neck region [19]. Cortical thickness in the tibia was also reported to have an association with aBMD and fracture incidence in postmenopausal people [20]. Furthermore, with high-resolution peripheral quantitative computed tomography, Helfen reported that the mean thickness of cortical bone in the SNR was decreased, and cortical porosity, is an essential factor explaining bone fragility and fracture risk in old patients, increased during aging [12, 21]. In accordance with those studies, the results in our study demonstrated that the cortical thickness in this cortical change region decreased with aging. The cortical thinning process can be accounted by the fact that the conversion of compact to cancellous bone arising from disordered Haversian remodeling and the trabecularization of the endocortical region [22–24]. It was also reported that 80% of all fractures in older individuals are located at sites that are mainly cortical and occur after the age of 60 years when the rate of trabecular bone loss decelerates [25, 26]. However, the reason why the fracture was primarily obvious in this cortical changing region rather than other cortical regions, such as the diaphysis, is still not completely understood.

Furthermore, the height of this region decreased from Group I to Group III, but surprisingly increased from Group IV to Group V. The relationship between age and height of the cortical change region was subject to curve-fitting and exhibited a shape similar to a parabolic relation. The valid height increase in older years can be explained by the fact that it was associated with osteoporosis, but the decreasing trend at a young age is worthy of further study. Therefore, the height and cortical thickness changes in this region are more complicated than we traditionally imagined, and some other mechanisms will affect the process of bone thinning and the remodeling process. In previous research, it was considered that age-related bone loss was found after a series of bony structural changes, and bone structure is preserved in mechanically loaded regions. In contrast, less mechanically loaded regions are attenuated with age [27, 28]. For example, the superior cortex of the femoral neck has been exhibited to become thinner much faster with age compared with the inferior cortex [29]. As a non-weight-bearing bone, cortical bone changes in the proximal humerus were more suitable to reflect the normal regulation process of osteoporosis without obvious external bearing force. Based on the increasing height of the cortical change region found in elderly subjects, it was considered that the region was the early phase where osteoporosis appeared, similar to the distal radius. The coalescence located between the epiphysis and diaphysis (age 16–18 years), which is located at the same place as the cortical change region, involved the last part of epiphyseal closure compared with the epiphysis of the greater or lesser tuberosity [30]. The resting zone maintains the growth plate grown by expressing parathyroid hormone-related protein, and it has an interaction with Indian hedgehog (Ihh) which was released from the hypertrophic zone [25, 26, 31, 32]. It was also reported that skeletal stem cells steadily formed in PTHrP^+^ chondrocytes which located at the growth plate resting zone [33]. Therefore, the resting zone of the growth plate might still exist after closure, and some skeletal stem cells or residual chondrocytes continue to regulate bone maturation until the age of 30 years old [33]. Along with the special position of the cortical change region, we would like to consider that the cortical changing region was formed after the metaphysical epiphyseal plate region closed in adults.

**Fig. 6:**
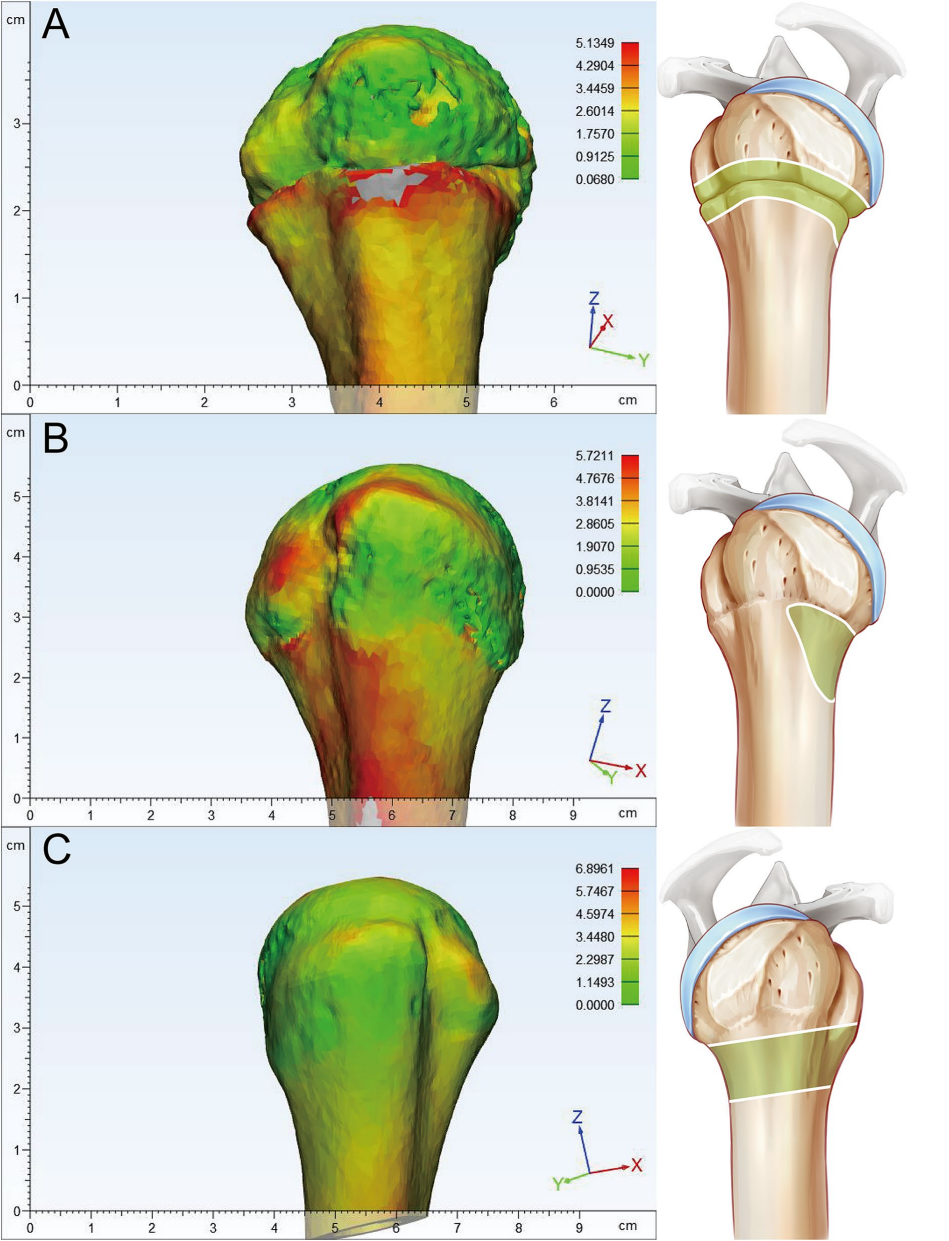
The cortical change region did not exist integrally in younger subjects. **A** cortical change region in an 8-year-old patient. **B** The cortical change region was partly formed in a 16-year-old male patient. **C** The SNR was completely formed in a 17-year-old female patient. Females were more inclined to mature than males

As the connecting region of the diaphysis and humeral head, the cortical thickness value of the cortical change region found in our research was thinner than the diaphysis, and thicker than the greater tuberosity. To further identify the character of this connected region, subjects younger than 18 years were enrolled, and an obvious boundary was noted between the greater tuberosity and diaphysis region in people younger than 18 years, and the cortical changed region did not or partly existed in these individuals (Fig. 6). A region with significant cortical thickness changes was only found when the proximal epiphyseal plate was closed. A precise definition of the SNR is lacking, and most authors defined it as a transition region of trabecular bone to cortical bone with a relatively thinner diameter. The SNR was always considered to have a high rate of fracture, and it was notable that the description of the SNR had many similar characteristics to this cortical change region. In our previous experimental results, the main fracture lines clinically diagnosed as surgical neck fractures were all located in the cortical change region, so it was believed that the SNR coincided with the cortical change regions, an area that was formed after the proximal epiphyseal plate closed at 18 years old. Therefore, it was reasonable to conclude that the surgical neck was a region that originated from or formed after the proximal humeral epiphyseal plate closed.

The limitation of the research was the number of subjects enrolled, and the morphology with different sexes, body anthropometric data, body weight, BMI were not compared. More volunteers will be enrolled in our following work, and the changing characteristics of the SNR will be observed more obvious in women than in men due to the relatively earlier maturation and menopause in adolescence or aging females respectively. In our subsequent research, a special molecular biological index that may exist in the resting zone of the epiphyseal plate will be searched. Further research will evaluate the changes in the biomechanical strength of the SNR and whether antiresorptive or gene treatment influence the bone remodeling processes or morphological characteristics of the identified SNR (proximal humeral epiphyseal plate region). Relative histopathology and gene editing technology will be conducted to test the hypothesis of the existence of skeletal stem cells in the closed epiphyseal plate region in adults.

In conclusion, this study identified an obvious decrease in cortical thickness with aging, and the height was curve fitted with aging in SNR. The characteristics of the SNR reflected that it might have potentially originated from the proximal epiphyseal plate closure, but need to be tested in further research.

## Data Availability

All relevant data are within the manuscript.
